# Acute kidney injury after aneurysmal subarachnoid hemorrhage: is chloride really responsible?

**DOI:** 10.1186/s40560-020-00492-x

**Published:** 2020-09-26

**Authors:** Gildas Gueret, Pierre Lefebvre, Pascale Le Maguet, Renaud Fabre

**Affiliations:** Service d’anesthésie, centre hospitalier de Cornouaille, 29000 Quimper, France

**Keywords:** Chloride, Acute kidney injury, Subarachnoid hemorrhage

## Abstract

Sadan et al. find an association between acute kidney injury and high chloride containing a hypertonic solution. Recent large prospective non-randomized studies bring conflicting results on the relationship between chloride and acute kidney injury. We discuss Sadan et al.’s results according to the recent literature.

Dear Sir,

We were interested in the article written by Sadan et al. [1]. After aneurysmal subarachnoid hemorrhage, the authors found an increase in acute kidney injury (AKI) for patients receiving high-chloride versus low-chloride containing a hypertonic solution. The authors concluded that NaCl/Na-acetate may modify the risk of AKI by reducing chloride load. Concerning intracranial pressure decreasing, both product’s effect was similar.

We do not fully agree with their conclusions.

First, the authors planned to randomize 60 patients (30 patients per group); however, only 32 were analyzed.

Then, the results seem to show that groups are not strictly similar despite randomization. In fact, cerebral edema in Na-acetate group could be less serious because they required a lower number of hypertonic solution doses. Moreover, the rate of AKI was lower in the NaCl/Na-acetate group as compared with the NaCl group, while the delta of creatinine post randomization was similar between groups. We only observe two patients who developed AKI after randomization, including one on the sixth day. It is probably too late to blame chloride load.

Third, some confounding factors are not indicated in the study. Intracranial hypertension, osmotic therapy, and vasopressor therapy are known to be risk factors of AKI after subarachnoid hemorrhage [2]. But in Sadan et al.’s study [1], we do not know how many patients received vasopressor therapy before and after randomization in each group. Likewise, before randomization, did the patients receive the same amount of hypertonic solution, and was intravenous chloride load similar in each group? In fact, in this study, delta intravenous chloride load and treatment with NaCl/Na-acetate appear as factors influencing renal prognosis.

Finally, in figure 3B, we observe seven patients of the 23.4% NaCl group who developed AKI. However, 53.3% of the 15 patients correspond to 8 patients.

Sadan et al. performed an interesting study, but several biases limit the author’s conclusions. Notably, AKI mainly occurred before randomization. It seems difficult to incriminate the type of solute and chloride load in AKI. On the other side, the responsibility of chloride in AKI is still discussed in large non-randomized controlled trials (RCT) performed in ICU [3], in operating room [4], and in RCT realized in high-risk surgical patients like kidney transplantation [5]. Large RCTs are needed to definitively conclude about the role of saline and chloride load in AKI [6].

## Abbreviations

RCT: Randomized controlled trials
AKI: Acute kidney injury
